# Selenium Deficiency Due to Diet, Pregnancy, Severe Illness, or COVID-19—A Preventable Trigger for Autoimmune Disease

**DOI:** 10.3390/ijms22168532

**Published:** 2021-08-08

**Authors:** Lutz Schomburg

**Affiliations:** Charité–Universitätsmedizin Berlin, Corporate Member of Freie Universität Berlin and Humboldt Universität zu Berlin, Institut für Experimentelle Endokrinologie, Cardiovascular–Metabolic–Renal (CMR)-Research Center, Hessische Straße 3-4, Charitéplatz 1, 10117 Berlin, Germany; lutz.schomburg@charite.de; Tel.: +49-30-450524289

**Keywords:** autoimmune thyroid disease, diabetes mellitus, Graves’ disease, Hashimoto thyroiditis, infection, inflammation, long-COVID, rheumatoid arthritis, selenoprotein P, sepsis

## Abstract

The trace element selenium (Se) is an essential part of the human diet; moreover, increased health risks have been observed with Se deficiency. A sufficiently high Se status is a prerequisite for adequate immune response, and preventable endemic diseases are known from areas with Se deficiency. Biomarkers of Se status decline strongly in pregnancy, severe illness, or COVID-19, reaching critically low concentrations. Notably, these conditions are associated with an increased risk for autoimmune disease (AID). Positive effects on the immune system are observed with Se supplementation in pregnancy, autoimmune thyroid disease, and recovery from severe illness. However, some studies reported null results; the database is small, and randomized trials are sparse. The current need for research on the link between AID and Se deficiency is particularly obvious for rheumatoid arthritis and type 1 diabetes mellitus. Despite these gaps in knowledge, it seems timely to realize that severe Se deficiency may trigger AID in susceptible subjects. Improved dietary choices or supplemental Se are efficient ways to avoid severe Se deficiency, thereby decreasing AID risk and improving disease course. A personalized approach is needed in clinics and during therapy, while population-wide measures should be considered for areas with habitual low Se intake. Finland has been adding Se to its food chain for more than 35 years—a wise and commendable decision, according to today’s knowledge. It is unfortunate that the health risks of Se deficiency are often neglected, while possible side effects of Se supplementation are exaggerated, leading to disregard for this safe and promising preventive and adjuvant treatment options. This is especially true in the follow-up situations of pregnancy, severe illness, or COVID-19, where massive Se deficiencies have developed and are associated with AID risk, long-lasting health impairments, and slow recovery.

## 1. Selenium and Selenoproteins

The trace element selenium (Se) is a micronutrient that is notable for several reasons, e.g., its essentiality, high medical relevance, and unique biochemistry [1]. It is part of the proteinogenic amino acid selenocysteine (Sec), for which an elaborate biochemical pathway has evolved and been preserved in many species [2]. Sec is synthesized on a seryl-loaded tRNA as a template, and it is used via one of two specific tRNA^[Ser]Sec^ isoforms [3,4], before being inserted during ribosomal translation into a small family of selenoproteins [5]. The distribution of Se to different organs and tissues, as well as to the different gene transcripts for selenoprotein biosynthesis, is hierarchically regulated and involves the two tRNA^[Ser]Sec^ isoforms [6,7,8]. Importantly, some selenoproteins are essential for life and are preferentially synthesized; inactivation of the respective genes in mouse models turned out embryonically lethal [9,10]. There is some overlap, but also certain distinctive differences to their roles and essentiality in humans [11]. A growing number of inherited diseases provide evidence for the distinctive roles played by selenoproteins in human health [12]. Hereby, the essentiality of Se is impressively underlined, as originally highlighted in 1957 by Schwarz and Foltz studying liver necrosis associated with vitamin E deficiency [13]. The tissue degeneration observed was efficiently prevented by supplemental Se [14], via increased biosynthesis of selenoenzymes [15]. It was only recently that the tight interplay among vitamin E, selenoproteins, and the accumulation of lipid peroxides inducing tissue degeneration and damage was identified as a conserved and tightly coordinated mechanism of cell death, referred to as ferroptosis [16]. These insights contribute to a better understanding of the molecular interrelation of Se and selenoproteins with energy metabolism and damage, as well as aging and decay, as underlying bases for the protective effects of Se in acute and chronic diseases [17].

### 1.1. Model Systems, Inherited Diseases, and Hierarchical Selenium Supply

Besides being essential for embryonic development, the identified inherited diseases with mutations in genes encoding selenoproteins or in essential factors involved in Se metabolism provide a more refined view on the essential role of selenoproteins [18,19,20]. The first identified example was a central component of the biosynthesis machinery, i.e., the RNA-binding protein SECISBP2 involved in correctly decoding the codon UGA for directing co-translational Sec insertion [21]. The affected children presented with an altered thyroid hormone pattern, reduced selenoprotein levels, and delayed growth that was not correctable by Se supplementation [22]. A more comprehensive characterization of additional young and adult subjects with mutation in *SECISBP2* indicated a spectrum of phenotypes, many of which resemble findings known from specific mouse models, e.g., delayed bone development, male infertility, metabolic dysregulation, elevated stress sensitivity and, importantly, disrupting effects on the immune system [23]. A detailed overview on the immune-relevant selenoproteins and their role in lymphocyte development, immune response, and effects on cytokine release and signaling can be found in excellent pieces of work published elsewhere [24,25,26,27,28,29,30].

The strong effects observed in the human patients with inherited defects indicate the extreme end of a spectrum of phenotypes that may result from insufficient selenoprotein expression. Certain single nucleotide polymorphisms (SNPs) in selenoprotein genes are associated with more subtle effects that may still be of relevance for normal development and human health, as seen in population-wide studies [31]. This line of research can efficiently study the full set of selenoprotein encoding genes along with additional components involved in Se metabolism and biosynthesis [20,31,32,33]. Notably, evolutionary selection of certain genotypes may have enabled a better adaptation to nutritional restrictions, and render subjects less sensitive to poor supply in a Se-deficient environment [34]. A full overview of the potential interactions between variations in Se-related genes and health issues can be found elsewhere [35,36,37]. Notably, certain genotypes associate with autoimmune disease (AID) risk and autoantibody titers, e.g., in selenoprotein S (*SELENOS*) [38] or selenoprotein P (*SELENOP*) [39].

Apart from genetics, the undisputed greatest influence on selenoprotein expression is the dietary supply of the essential trace element, which varies widely around the world [40]. Under insufficient supply, the hierarchical principles that govern the targeted Se distribution within the organism protect essential tissues and biochemical pathways from deficiency [6,7,8]. A poor Se status of a given subject therefore does not cause an obvious phenotype or detectable health symptoms, and usually goes unnoticed if no laboratory analysis is conducted. Nevertheless, large analytical studies indicate that a Se supply below the recommended intake levels is associated with increased disease risks, especially for subjects with chronic disease, inflammation, or other predispositions [40,41]. It is time to take this knowledge more seriously and to realize that Se deficiency is difficult to detect, but easily avoidable. The essentiality is similar to, e.g., iodine, where population-wide nutritional measures and suitable supplements yield remarkable positive health effects [42]. In the case of iodine, deficiencies are visible and better known. Until now, meaningful measures to correct a poor Se supply have rarely been taken by the authorities. Finland is a remarkable exception and decided more than three decades ago (1984) to take action, and to add supplemental Se systematically to the fertilizers used in agriculture [43]. This countrywide supplementation program was successful in increasing the average Se intake of the Finish population to a likely health-supporting level [44]. Unfortunately, a control population is not at hand and direct health-supporting effects are difficult to be deduced, while positive effects on animals as well as plants are recorded [45]. A sufficiently high Se intake will improve general selenoprotein expression levels and raise the Se status in particular in those tissues that are stringently dependent on the Se supply, i.e., those that are low in the hierarchical supply, including liver, muscle, the gastrointestinal tract, and the immune system [24,46]. A sufficiently high Se status supports their integrity, metabolic activities, and responsiveness to regulatory signals, while Se deficiency compromises the adequate functioning of these organ systems and reactive cell types.

### 1.2. The General Role of Se Status and Supplemental Intake for Human Health

A Se deficit alone does not lead to an obvious phenotype, but it predisposes to certain diseases. The health risks of a relatively low Se intake become apparent in observational or intervention studies when biomarkers of Se status are analyzed in relation to disease prevalence and incidence. Using dietary Se intake data for deducing Se status has proven most difficult, as the same food items vary strongly in Se content in relation to the origin of production [40,47,48,49,50,51]. Among the most suitable biomarkers of Se status are total Se concentrations in serum or plasma and circulating selenoproteins [52,53,54,55,56]. These biomarkers are applied to epidemiological or intervention studies where only small amounts of biosamples are available. Unfortunately, only a few studies have determined the Se status by assessing more than one biomarker, or tried to closely monitor changes in Se status prior, during and after an intervention [41]. This constitutes a shortcoming in many fields of nutrition research, and unfortunately also in the studies on Se in AID.

The number of well-controlled and sufficiently powered randomized control trials (RCT) with supplemental Se is small, and the largest and most comprehensive ones have been conducted in the USA, where a large fraction of the population exhibits a replete Se status [57]. This is in contrast to the majority of subjects residing in other parts of the world [58]. The fortunate situation in North America is mainly due to the high Se content in the soils used for agriculture. The scientific strength of the large US trials for the role of Se in human health is therefore largely restricted to the issue of pharmacological Se effects, i.e., questions of side effects and toxicity in sufficiently supplied subjects [41,48]. This notion is best exemplified in the field of chemoprevention, where two large RCT with supplemental Se have been conducted over long time-periods. In the Nutritional Prevention of Cancer (NPC) study, roughly two thirds of the participants had a high baseline Se status [59], whereas in the Selenium and Vitamin E Cancer Prevention Trial (SELECT) follow-up study, almost all of the enrolled subjects were sufficiently supplied with Se [60]. Accordingly, chemopreventive effects were observed in the small fraction of Se-deficient participants in the lowest tertile of baseline Se in NPC only [61].

**Figure 1:**
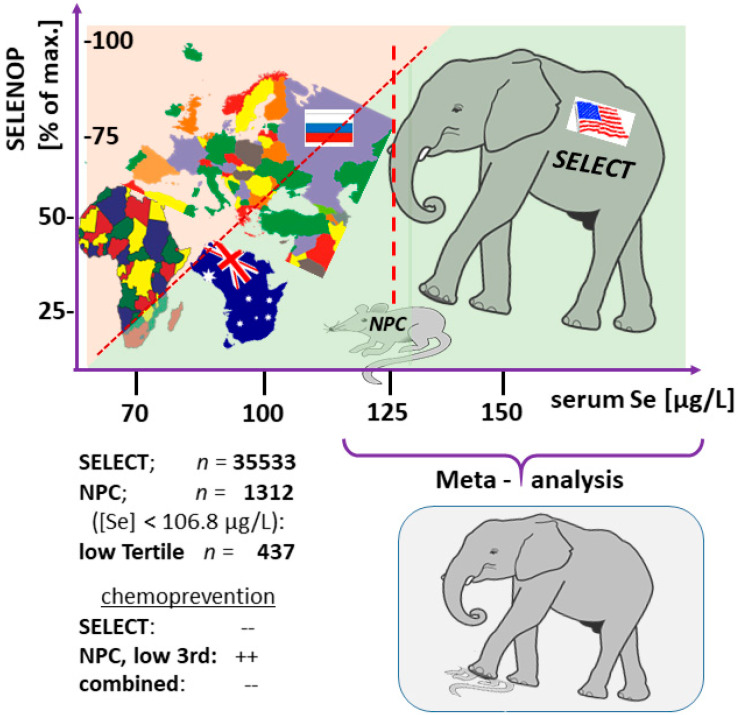
Importance of baseline Se status for the interpretation of RCT results. Dietary intake of Se varies widely in different parts of the world, and large parts of, e.g., the USA, are well supplied. This is in contrast to many other countries. A saturated expression of the Se transporter selenoprotein P (SELENOP, (% of max.)) indicates a sufficiently high Se status and corresponds to serum Se of 120–130 µg/L (broken line). As only one third of participants in the NPC study were below this threshold and showed positive health benefits from supplemental Se, their contribution to the overall effects is diluted and lost in meta-analyses. Positive health effects of supplemental Se can only be expected if selenoprotein expression is affected, and studies performing substitution versus supplementation need to be distinguished.

When summarizing both studies, it is inappropriate to combine the trials into one meta-analysis, as subjects with suboptimal Se status were under-represented, i.e., around 1–2% only when the studies are aggregated (Figure 1). From today’s viewpoint, correcting a Se deficit is a meaningful health-supporting measure with biochemical effects, whereas supplementing well-supplied subjects with additional Se appears unnecessary [41,62,63]. Consequently, meta-analyses on supplemental Se by combining studies that differ profoundly in baseline Se status fail [64]. Supplementing subjects beyond their needs may be conducted as experimental attempts of targeted therapy, e.g., in oncology, where exceedingly high pharmacological Se dosages are studied for death-inducing effects on tumor cells [65,66,67]. However, no meaningful insights into the role of Se deficiency for the immune system and AID risk can be expected from these approaches.

### 1.3. Uneven Worldwide Clinical Research on the Role of Se for Human Health

**Figure 2:**
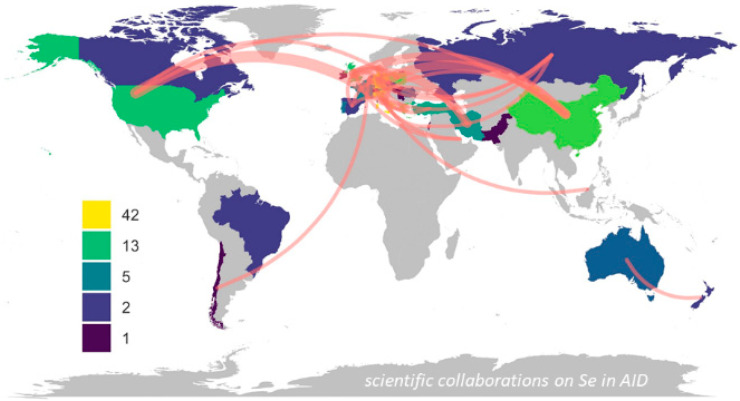
Overview on international collaborations on a potential role of Se in autoimmune disease. Countries with prevalent Se deficiency are particularly active in this line of research. The red lines indicate collaborations as published in the Web of Science (accessed on 4 April, 2021), with line thickness corresponding to the number of entries. The countries are color-coded for the number of contributions, with Denmark, Germany, and Italy yielding highest marks (yellow). Analysis was conducted for references matching the term “ALL = (selenium autoimmune thyroid)”, using the ‘bibliometrix’ and ‘ggplot2’ packages in R (Version 2.1.0, R: A Language and Environment for Statistical Computing; The R Foundation for Statistical Computing: Vienna, Austria, 2020).

The excursion on supplemental Se and well-controlled large RCT may seem of little relevance to Se in AID, while in fact the knowledge and understanding of this background is of central importance for the interpretation of the current database and the general appreciation of Se in health and disease. It is almost impossible not to mention these two contradictory RCT when discussing the perspectives of Se in the ambulant and clinical care of patients. While the large and expensive SELECT study failed to achieve its primary goal of prostate cancer prevention, its scientific quality and technical rigor was undoubtedly of the highest order and provided insights into the human organism’s tolerance to high Se intake. However, its relevance for the risks from Se deficiency and the importance of correcting a pre-existent Se deficit for preserving health and reducing disease risks is marginal. Despite these limits, these particular RCT are constantly referred to when Se supplementation trials are planned, discussed, and neglected, analytical research is considered and then dismissed, or when funding is applied for and then finally denied. The failure of SELECT has not served the basic and clinical research on Se well. A thorough understanding of the reason for failure of this RCT also helps to understand why smaller analytical and intervention studies can still be of profound scientific value, when conducted in populations of low or moderate Se status under high scientific standards. This is another peculiarity for Se in medicine; many important and meaningful studies are conducted in areas with Se deficiency [68,69], which explains the uneven distribution of collaborations on Se in AID, highlighting in particular central Europe, China, Turkey, and Iran as exceptionally active regions (Figure 2).

The concept of “substitution” versus “supplementation” may be introduced here, with the former indicating a targeted supply in order to correct a nutritional deficit, while the latter indicates pharmacological supply on top of a sufficient baseline status [41,70]. The chemopreventive effects of Se in the NPC study were restricted to the intake as substitution, since the responsive subjects were residing in the lowest tertile of baseline Se status, where full expression of selenoproteins was not yet achieved [71,72]. This setting is in contrast to the unsuccessful SELECT intervention, where Se was applied on top of a sufficiently high baseline Se status as a pharmacological supplementation [48,62]. Consequently, when we follow the same hypothesis and assume that a Se deficit constitutes a risk factor for AID, we need to focus on trials with Se-deficient subjects residing in areas of general poor Se supply. The published evidence for a potential role of Se in AID needs to be interpreted with this distinction in mind, and null results from areas with high baseline intake are unlikely of value for this issue. Given the inconsistent Se status around the world, no coherent and uniform guidelines from the various national expert committees on Se substitution can be expected, and recommendations on supplemental Se will differ with good cause between areas with low and high habitual Se intake.

## 2. Clinical Studies Linking Se Status and Autoimmune Diseases

Clinical studies on Se are either observational and compare Se status between diseased and healthy subjects or in relation to disease severity, or trials are interventional, supplying extra dosages like in the aforementioned RCT that used a daily supplement of 200 µg Se, either as Se-rich yeast (NPC) or selenomethionine (SELECT). As mentioned above, analytical studies relying on dietary Se intake are difficult to conduct; therefore, biomarkers of Se status are analyzed [51]. To this end, three biomarkers have achieved high acceptance in the field, i.e., the enzymatic activity of the extracellular glutathione peroxidase-3 (GPX3), total serum or plasma Se concentration (total Se), and the circulating transporter selenoprotein P (SELENOP) [54,71,73]. Some analytical studies have combined more than one biomarker (or determined Se content in hair or nails in parallel to blood), and yielded some congruent and meaningful results [53,74]. The published large RCT mentioned have unfortunately analyzed only one Se status biomarker.

### 2.1. Selenium Status and Hashimoto’s Thyroiditis

**Figure 3:**
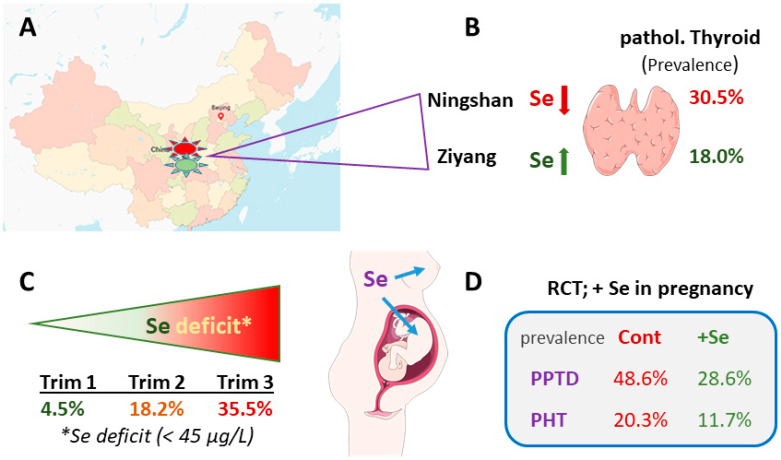
Importance of Se deficiency as druggable risk factor for AID. (**A**) High thyroid disease prevalence (pathol. Thyroid; goiter, (sub-) clinical hypothyroidism or autoimmune thyroiditis) is observed under low baseline Se intake. (**B**) The population on low Se intake (Ningshan) had an almost twice-higher incidence of thyroid disease as compared to the population with higher Se intake (Ziyang). (**C**) During pregnancy, Se status in the pregnant mothers decline from trimester 1 (Trim 1) to Trim 3, causing a severe Se deficit (<45 µg/L) in one-third of pregnancies in a European observational study. (**D**) Supplemental Se during pregnancy was capable of suppressing the high incidence of postpartum thyroid dysfunction (PPTD) and permanent hypothyroidism (PHT) in predisposed women ca. two-fold.

Hashimoto’s thyroiditis (HT) is a highly prevalent, renowned, and dynamic AID, characterized by autoreactive lymphocytes invading the thyroid gland. The immune process causes swelling, initial signs of hyperthyroidism and malaise, followed by a long and often chronic phase of progressive gland destruction, hypothyroidism, and increasing need for thyroid hormone replacement therapy [75]. The incidence is high and on the rise, exhibits individual disease courses and it is more prevalent in women than in men [76]. Observational studies in the EU have indicated that Se deficiency is positively associated with thyroid gland volume and nodule formation [77,78]. Conclusive evidence for a causal role of Se deficiency in promoting hypothyroidism and autoimmune-related thyroid gland damage (i.e., chronic stages of Hashimoto’s thyroiditis) has come from a large cross-sectional study in China enrolling >6000 subjects. The participants were residing either in a Se-deficient area (Ningshan) or in an area with moderate Se status (Ziyang) [79]. Median plasma Se concentrations differed two-fold (104 vs. 57 μg/L), i.e., from sufficient for a saturated expression of most selenoproteins to a moderate deficiency. Accordingly, thyroid disease prevalence was almost twice as high in the Se-deficient area of Ningshan as compared to Ziyang (Figure 3A,B). The findings highlight that chronic Se-deficiency enhances the risk for AID of the thyroid, despite the notion that the populations were accustomed to their general Se supply [34]. Summarizing the different observational studies at hand, it becomes apparent that Se deficiency is a risk factor for increased thyroid gland volume, hypothyroidism, HT, thyroid nodules and associated health problems, and a population-wide approach as taken in Finland would likely reduce AID incidence and disease load in many areas of the world. Sex-specific differences in relation to the role of Se in goiter or HT incidence were reported from a large study in Europe [77], but were not observed in the large Chinese study [79]. Baseline iodine supply may modify the interaction of Se and thyroid disease [80], as Se status and goiter development are particularly interrelated under iodine deficiency [78,81]. Besides differences in the dietary Se intake, certain acute conditions are also associated with higher Se demands or increased Se loss, including severe disease, infection, or pregnancy. During pregnancy, increasing amounts of Se are transferred from the mother to the growing fetus, leading to an increasing deterioration of the pregnant woman’s Se status if the increased need is not counteracted by additional Se intake [82,83]. The deficit may even worsen thereafter due to lactation (Figure 3C), potentially eliciting negative health effects on both the child and mother under limiting Se supply [84]. After pregnancy, postpartum thyroid disease (PPTD) constitutes a frequently found AID [85], in particular for women with positive thyroid autoantibodies (aAb). In view of the developing Se deficit in pregnancy, a highly interesting and instructive RCT from Italy tested supplemental Se to prevent PPTD and permanent hypothyroidism (PHT) [86]. To this end, pregnant women with or without positive TPO-aAb were enrolled and monitored. The intervention was conducted with 200 µg of selenomethionine per day, and it was successful [86]. The incidence of both PPTD and PHT was reduced by almost twofold, and no side effects were noted (Figure 3D).

### 2.2. Selenium Status and Graves’ Disease

Graves’ Disease (GD), the second major AID of the thyroid gland, occurs when the aAb mimicking thyroid-stimulating hormone (TSH) directly activates the TSH-receptor, resulting in an overactive endocrine gland that is not controlled by negative feedback regulation and, thus, causes clinical symptoms of hyperthyroidism [87]. Like HT, incidence and prevalence of GD show a strong female-biased sex-specific difference [88]. The relation between Se status and GD is more complex and less-well understood in comparison to Se and HT [89,90], probably due to the positive effects of thyroid hormone on Se status and hepatic SELENOP biosynthesis [91]. Interestingly, no general interrelation between habitual Se intake and prevalence of hyperthyroidism was observed in the aforementioned large cross-sectional study in the two Chinese provinces with different Se intake and status [79]. However, a remarkable difference in GD prevalence was apparent when the data were analyzed for males and females separately; male subjects only seemed to react sensitively to Se deficits, and exhibited a higher prevalence of GD under low Se supply as compared to men with higher baseline Se intake, while disease prevalence of women remained largely unaffected [92]. Accordingly, the female-to-male ratio of hyperthyroidism (mainly due to GD) was 1.6 in the Se-poor area and 4.2 in the area with higher baseline Se intake (Figure 3B). This finding was statistically significant and completely unexpected, as it is widely assumed that sex-specific differences in AID incidence and prevalence are rather related to (epi-)genetic [93,94] or hormonal effects [95,96], like dysregulated X-chromosome inactivation [97] or estrogens and estradiol receptors [98,99], but not to micronutrient intake, again highlighting a peculiar sex-specific oddity of Se for the immune system. The surprising notion that a particular micronutrient, such as Se, which is associated with a number of sexual dimorphic effects in medicine and biology [7], is also strongly modifying the sex-specific risk for a prominent AID, opens a new and challenging perspective on the interplay of nutrition, genetics, and AID risk. Besides a number of analytical studies that provide a partly overlapping (but largely inconclusive) overall picture on the importance of Se for GD, one notable RCT reported remarkable positive health effects in GD-associated eye disease, i.e., Graves’ orbitopathy (GO). Proptosis of the eye ball and inflammatory markers were successfully suppressed and eye motility and quality of life were strongly improved by daily Se supplementation over six months in patients with mild GO [100]. The positive health effects lasted beyond study termination, but the underlying mechanisms are unknown, and the promising findings have not been replicated yet. Still, the results are taken seriously and Se is recommended in mild GO by leading experts in the field [101,102].

### 2.3. Selenium Status and Type 1 Diabetes Mellitus

Type 1 diabetes mellitus (T1DM) is also known as “insulin dependent diabetes mellitus” or “juvenile diabetes”, and describes the autoimmune form of diabetes that mainly develops at a young age [103], but also occurs as a slowly developing latent autoimmune diabetes in adults (LADA) or as classical and abrupt adult-onset T1DM [104]. Disease course involves a progressive destruction of insulin-producing pancreatic beta cells, sooner or later necessitating insulin replacement therapy due to gland insufficiency, eventually along with other medication [105]. It is estimated that around one in ten cases of diabetes mellitus are due to the destructive AID, with certain population-specific differences, while the majority are associated with insulin insensitivity of target tissue, being overweight, and often older age, typically denoted as type 2 diabetes mellitus (T2DM) [106]. Recent research indicates that diabetes mellitus should be categorized more precisely, as overlapping and distinct phenotypes and disease courses require personalized analyses and treatment regimens [107,108,109,110,111]. Despite the high prevalence and rising incidence of diabetes mellitus along with the essential role of Se for the endocrine and immune system, research activities on Se in the AID form of diabetes (T1DM) are sparse.

This is in contrast to some knowledge on the interrelationship of Se with T2DM, where an increased serum or plasma Se concentration is observed in many patients [112,113,114]. The dysregulation involves insulin resistance of liver, and its positive effect on hepatic SELENOP biosynthesis, causing higher serum Se concentrations [41,115,116]. Conversely, Se deficits are associated with hypoglycemia, i.e., a dysregulation of blood glucose levels in the opposite direction [117]. In contrast to initial reports, the results from large and well-controlled RCT indicate that Se supplementation is not causally related to T2DM incidence, not even in Se replete populations [41,118,119]. In comparison to T2DM, the data on Se and T1DM risk and course are few. In children, a small case-control study indicated a slightly increased serum Se status in the pediatric patients with T1DM as compared to controls (74 ± 8 vs. 65 ± 8 µg/L) [120]. In a larger study, an inverse relationship was observed, which was especially pronounced in children with T1DM and poorly controlled blood glucose, who displayed reduced serum Se and relatively low erythrocyte GPX1 activity [121]. This finding accords with a report on relatively low Se in erythrocytes of children with T1DM [122]. A case-control analysis using siblings of diseased children observed no difference in relation to GPX activity between T1DM and controls [123], in agreement with a small study on adults from the UK, where plasma Se was comparable between patients with T1DM, T2DM, and controls [124].

From these few studies, a picture emerges with two opposing forces apparently influencing the relationship of Se with diabetes mellitus; both the autoimmune nature of T1DM and the increased fat mass in many patients with T2DM are associated with an activated immune system, inflammation, and increased cytokines/adipokines including IL-1 and IL-6 [125,126]. This condition is known to impair hepatic selenoprotein biosynthesis and SELENOP expression [127,128,129]. At the same time, insulin resistance and increased glucose availability exert positive metabolic effects on hepatic SELENOP biosynthesis, consistently in T2DM and with an age- and disease-stage dependence in T1DM, compatible with a generally increased Se status in T2DM, and an unpredictable relationship in T1DM. In Finland, it was observed that the diet could influence the interrelation between Se status and disease in T1DM. Children with T1DM had a higher Se status as compared to age-matched controls before the nation-wide Se supplementation was in place. This difference may have been due to a better food quality higher in Se content selected by the patients and their well-caring parents. However, the difference in serum Se disappeared between T1DM and control children with the population-wide supplementation and a general increase in Se intake [130], indicating that the potential advantage of choosing high quality food items in T1DM was crucial and decisive at times of poor Se supply, but became dispensable in face of the universal enrichment of Finish food with Se. This notion underlines again the particular relevance of population-wide measures for improving Se status under poor habitual Se availability.

### 2.4. Selenium Status and Rheumatoid Arthritis

Rheumatoid arthritis (RA) describes a third major group of inflammatory and chronic AID affecting primarily the joints, but eventually also spreading to remote sites including skin, kidneys, nerve tissue, lung, heart, and other organ systems [131]. The disease activity shows some circadian rhythm, and it can precipitate in sporadic longer-lasting waves of increased or decreased activity in a very personalized manner. In general, RA constitutes a constant and increasing threat to joint function, movement, quality of life, and overall health [132]. In agreement with other AID, its prevalence is higher in women than in men, with a disease peak in late adulthood [133]. Serum Se concentrations were found decreased in a set of 101 patients with seropositive RA compared to controls, even among US patients with high baseline Se concentrations exceeding the levels needed for full expression of circulating selenoproteins (148 ± 42 vs. 160 ± 25 µg/L) [134]. In a study on patients suffering from a rare and severe form of RA, i.e., systemic sclerosis, all three biomarkers of Se status (GPx3, total Se, and SELENOP) were significantly decreased as compared to controls [135]. This tendency was also reported in a meta-analysis from 2016, highlighting a relatively low Se status in patients with RA [136]. The deficiency is obviously not restricted to the trace element Se, but can also be observed for a second immune relevant micronutrient, i.e., zinc (Zn) [137]. In parallel, the trace element Cu seems to be slightly elevated in serum of RA patients, consistent with the positive acute phase reaction of liver-derived ceruloplasmin as systemic Cu transporter [138,139].

The imbalance in trace element concentrations underlines the chronic inflammatory nature of RA and an elevation of pro-inflammatory cytokines, with IL-6 and IL-1ß likely taking center stage for the observed effects on the micronutrient status [140,141]. Notably, the interaction between Se deficiency and enhanced inflammation is not necessarily unidirectional and self-limiting, but rather constitutes a vicious cycle with self-amplifying characteristics, similar to the situation in sepsis [142]. The Se status is known to affect NF-kB activity, and a reduced Se status will cause an up-regulation of a whole set of inflammation-relevant genes [143]. During NF-kB activation, and in response to other noxae, a number of intracellular stress-regulated selenoproteins are induced including essential components affecting ER quality control mechanisms like SELENOF, SELENON, SELENOS, SELENOT, or SELENOV [144,145,146,147,148]. Notably, SNPs in the promoter of *SELENOS* have directly been associated with IL-6, IL1ß, and tumor necrosis factor-alpha expression in human subjects [149]. Accordingly, an interaction of *SELENOS* genotype and IL-1 was identified for RA risk [150]. The potential molecular interactions are supported by recent clinical data. Anti-rheumatic treatment showed a sustained positive effect on Se concentrations [151]. The increase was parallel to a reduction in inflammatory activity and suppression of pro-inflammatory signaling [151]. Collectively, the chronic inflammatory nature of RA seems to directly impair Se metabolism, with a negative effect on hepatic SELENOP biosynthesis and consequently a reduced serum Se status in patients as compared to healthy controls, which closes a self-sustaining but druggable feedforward pathway [152].

Some pilot intervention studies with supplemental Se have been conducted, albeit with small groups of RA patients only, short duration, and a limited monitoring of changes in the important biomarkers of Se status. An increase in GPX activity to control levels was observed in a six months Se supplementation trial in a group of six patients in relation to six control subjects as early as in 1987 [153]. An RCT using 200 µg Se per day as selenized yeast for 90 days reported an increase in serum Se concentrations and an improvement in several parameters of RA, including quality of life [154]. However, the positive effects were observed in both study arms and a specific health impact of the trace element exceeding the placebo effect was not detected with statistical significance. Collectively, the clinical experience with Se in RA is very rudimentary in view of the importance and high prevalence of this potentially devastating AID. Supplemental Se may protect from severe disease course, in view that RA is associated with progressive Se loss, and supplemental Se may counteract efficiently the decline. It is not known why this cost-efficient and safe adjuvant treatment option is not explored in sufficiently sized and well-controlled RCT, as the chronic, severe, and progressive nature of RA serves as a paradigm for an inflammatory, most prevalent and relevant AID, which affects millions of people, in particular young and adult women, and is responsible for many lost years of quality of life.

## 3. Correcting Se Deficits in Prevention and Treatment of Autoimmune Diseases

The majority of analytical studies indicate a profound Se deficit in patients with an AID, likely linked to disease activity, ongoing inflammation, and an elevated activity of an autoreactive immune system. It is also known that a sufficient Se supply is needed to support and moderate the activity of immune cells and to avoid an overshooting immune response [27,39,129,155]. Consequently, it appears plausible that a Se deficit aggravates AID course, and supplemental Se may elicit positive health effects, contribute to the normalization of the immune response, reduce autoreactive disease activity, and support other therapeutic measures in an adjuvant mode.

Several supplementation studies have been conducted, albeit mostly with small numbers of patients and for short periods of time only. Comparing the three types of AID mentioned above, the majority of intervention studies with supplemental Se have addressed the two autoimmune thyroid diseases, i.e., HT and GD [89,90]. Despite an initial enthusiasm, the overall picture is ambiguous as several trials have reported positive effects, but a number of similar studies reported no health improvements [89,90]. Negative side effects have not been observed. At present, the major reason for these equivocal results is unknown, as neither the nature of the most suitable selenocompound nor the optimal treatment duration, nor best dosage have been specified. Similarly, the ideal baseline Se status and best time for treatment initiation are not identified. The paucity of knowledge is partly due to the small groups studied, the heterogeneous groups of patients, and a lack of detailed monitoring of Se status at baseline, during supplementation and at study end. In order to resolve some of these inconsistencies, two well-controlled intervention trials with Se in patients with HT and GD, respectively, are under way and will hopefully shed some more light on the potential efficacy of supplemental Se on disease severity and course, as well as on the underlying reasons for the contradictory experiences. The chronic autoimmune thyroiditis quality of life Se trial (CATALYST) has enrolled almost 500 patients, and the effects of supplemental Se (200 μg per day) or placebo on the disease-related quality of life along with changes in thyroid aAb concentrations during an intervention period of one year are evaluated [156]. The GRAves’ disease Selenium Supplementation (GRASS) trial is of similar size and design, and primarily studies the effects of Se on anti-thyroid drug treatment failure [157]. Importantly, both studies are conducted in Denmark, i.e., with European patients of insufficient baseline Se status for full expression of selenoproteins. Until the results from these well-designed and promising RCT are at hand, the data collectively indicate that dosages up to 200 µg Se per day, as also used in the aforementioned large RCT in the USA, do not harm and may reduce inflammation and autoantibody concentrations in AID.

## 4. Immune Dysfunction in Se Deficiency–Possible Molecular Mechanisms

Lymphocytes and the immune system show certain functional alterations in Se deficiency and upon Se substitution, indicating that their position within the hierarchical order of supply with the essential trace element is not at the top, where Se status is maintained even in low supply, e.g., the endocrine or central nervous system [7,24,29]. However, Se supply beyond the needs for saturated expression of selenoproteins shows little if any effects on the immune system, underlining the essential role of selenoproteins and their adequate expression for a regular functioning of lymphocytes [158]. Declining Se status in disease or pregnancy will consequently impair regular selenoprotein biosynthesis and regular immune system function. Accordingly, the members of the selenoprotein family with proven relevance for lymphocyte activity along with those having a general role in antigen processing and MHC-dependent presentation are the prime targets of Se substitution, and constitute the main culprit for immune dysfunction in Se deficiency [29,159]. The biochemical and physiological pathways affected by suboptimal expression of particular selenoproteins range from a poorly controlled intracellular peroxide and redox tone, failing quality control of newly synthesized proteins in the ER, impaired Ca signaling to an excessive oxidation of cellular components and death by ferroptosis (Table 1).

It is at present unknown which of these immune-relevant selenoproteins respond most sensitively to Se deficiency and constitute the rate-limiting factors for a dysfunctional immune system under low Se supply. Besides the selenoproteins that affect lymphocyte activity, migration, proliferation, survival, and interaction directly, a large number of selenoproteins is involved in quality control of newly synthesized proteins in the ER affecting correct protein folding and retrotranslocation of misfolded proteins into the cytosol for degradation [170]. Consequently, a disease- or pregnancy-induced decline of the Se status into a critical zone where these selenoproteins are not synthesized any more to the required expression levels will increase the risk for lymphocytes loosing self-tolerance and at the same time for a widespread presentation of wrongly processed novel autoantigens due to failing quality control in the ER. This hypothesis is compatible with many preclinical and clinical studies on autoimmune thyroid disease [89], but lacks solid and more comprehensive clinical data from the other prevalent autoimmune diseases, such as RA or T1DM, as mentioned above.

## 5. Potential Toxicity of Supplemental Se

A critical evaluation of the role of Se in AID needs to consider the potential toxicity of supplemental Se. The biological activity of Se as an essential trace element is mainly exerted via its incorporation into Sec-containing selenoproteins. High Se supply in excess of the required amounts can be toxic due to poorly characterized molecular effects [171], and in relation to the molecular form of the selenocompound ingested and its metabolic fate [67,172,173]. An incidence of endemic Se-related poisoning (“selenosis”) was observed in the naturally Se-rich Enshi county in Hubei Province, China, where the Se intake peaked to about 5000 µg per day due to the usage of stony coal of extremely high Se content [174]. In comparison, the recommended intake of Se for healthy adults in Austria, Germany, or Switzerland ranges at 60–70 µg per day, i.e., around one percent of these amounts [175].

A second relevant risk for selenosis is encountered when using Se-containing supplements that have been prepared with a lack of appropriate quality control. The consequences from this type of error were impressively documented in veterinary medicine, where wrongly formulated preparations caused several incidents of animal poisoning, e.g., sudden death of polo ponies upon receiving excessive amounts of supplemental Se causing serum concentrations exceeding 1000 µg/L [176]. Similar incidences are also known from human subjects consuming misformulated dietary supplements [177]. However, humans seem to be relatively robust to acute Se intoxication, as most of the symptoms described from a daily intake of pills containing 22–32 mg of Se per serving were reversible, and luckily, no fatal course occurred [177]. Even more surprising are sporadic case reports, e.g., from a pregnant mother taking 200 mg of Se per day (i.e., 1000-fold higher than the commonly accepted maximal dosage used in clinical trials) during gestational weeks 7 to 12 [178]. The women lost hair and fingernails, as expected from severe selenosis, but surprisingly gave birth at term to a healthy child [178]. These examples indicate that supplemental Se can be toxic and even fatal, irrespective of the underlying motivation for health-support or suicidal intention [179]. However, the dosages causing acute selenosis are far higher than the recommended amounts applied in clinical studies or provided by high-quality supplements, where up to 200 µg Se per serving per day proved safe, irrespective of the selenocompound used. The health risks from chronic oversupply are however poorly characterized, potentially due to some adaptation of the population [34].

## 6. Se-Deficiency as Potential Trigger of Autoimmune Disease

The hypothesis that severe Se deficiency contributes to AID risk as a trigger factor is supported by a limited body of evidence, but appears reasonable and scientifically congruent. The immune system is constantly balancing its activity for reliably recognizing self, responding to non-self and identifying variants resulting from malignant transformation, chemical modification, infection or mimicry [180]. A number of highly specialized immune cells help maintain this delicate balance and prevent AID. In order to work reliably, essential vitamins and trace elements are needed, and deficiencies are established risk factors for infections and inappropriate immune responses [181]. Several selenoproteins are known to contribute to ER quality control, intracellular calcium signaling, and antigen presentation, as well as to immune cell activation, suppression, proliferation, and differentiation [182]. Insufficient supply with trace elements needed interferes with these pathways, in particular in disease and under certain demanding conditions.

**Figure 4:**
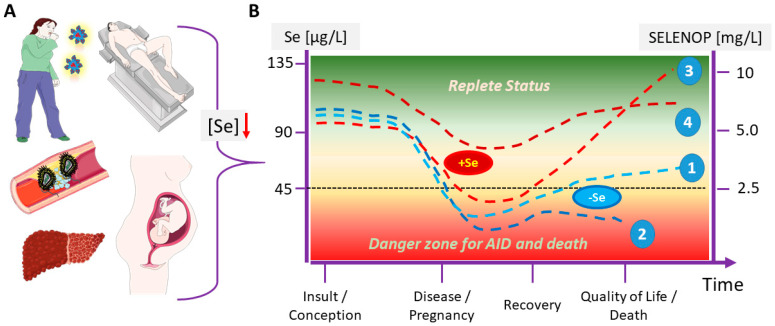
Hypothesis on Se decline into a critical zone as trigger for immune system failure, AID development, or even death. (**A**) Bacterial or viral infections, acute or chronic illness, AID, surgery, liver disease, or pregnancy are associated with a vicious cycle of inflammation, increasing cytokine levels and decreasing Se status. During and following to these conditions, AID may develop. (**B**) A disease-related drop in Se status below a certain threshold into a critical concentration range (“danger zone”) impairs regular immune system function and potentially disrupts self-tolerance, leading to AID. (1) Under regular conditions, disease-associated Se decline is transient, recovering with time. (2) Fatal disease course is associated with strong Se status decline and lack of its recovery. (3) Supplemental Se (+Se) reduces both the Se trough and time spent in severe deficiency, thereby likely improving odds of convalescence. (4) The risk for dropping into the danger zone of severe Se deficiency and immune system failure can be reduced by early Se supplementation, thereby starting on a sufficiently high status, ideally in combination with adequate monitoring in order to provide the amounts necessary and avoid side effects, i.e., to substitute what is needed without supplementing beyond requirement.

Bacterial and viral infections, acute and chronic inflammation, trauma, burn injury, childbirth, and surgical interventions are clinical conditions causing a declining Se status, as shown both in model systems and clinical studies alike (Figure 4A). On a molecular level, the downregulation of hepatic SELENOP biosynthesis by pro-inflammatory cytokines seems central for the observed decline in serum Se concentrations [183,184,185,186]. In parallel, the specific Se transport via SELENOP to target tissues becomes disrupted, and a systemic Se deficit may develop, in particular when baseline Se status is already low [8]. Notably, declining Se status and increasing cytokine concentrations are closing a feed-forward regulation, i.e., a self-amplifying loop [129,142,158]. The clinical conditions causing a decline in Se status show a considerable overlap to potential triggers for AID development, and both processes may be causally linked, i.e., acute Se deficiency may constitute an as-yet poorly appreciated trigger for AID development (Table 1). A decline below a certain threshold may tip the balance from regular lymphocyte function to disruption of self-tolerance, triggering autoreactive processes (Figure 4B).

Among the major modifiers of this process are the baseline Se status, before and during the early stages of disease, the severity, and course of the condition that is causing the Se decline along with the minimal Se concentrations that are reached during the disease. Supportive adjuvant therapy including supplemental Se will counteract the drop into the dangerous zone of severe Se deficiency (Figure 4B). Besides potentially triggering AID development, a severe Se deficit has been identified as mortality risk factor in severe illness, e.g., sepsis, COVID-19, liver disease, along with other critical conditions [187,188,189,190]. Notably, a sufficiently high baseline Se status is capable of preventing an uncontrolled and exaggerated immune response [149,191], with some sex-specific characteristics [7,129], and high potential in the prevention of severe COVID-19 [192]. The principle of positively affecting the immune system and decreasing inflammation by Se has just been verified in COVID-19 with patients displaying severe acute respiratory distress syndrome, where early supplementation was capable of restoring Se status [193].

In view that severe COVID-19 causes very strongly decreasing Se status, high mortality risk, and newly developing autoimmunity, it may also be hypothesized that severe Se deficiency may be related to post-acute sequelae and long-COVID symptoms [194,195]. It will be of high importance to monitor Se status during the current pandemic in both mildly and severely affected patients, and to delineate Se status decline and recovery to long-term health issues including autoimmune reactions to peripheral and central antigens. The author is convinced that supplemental Se to subjects with proven or predicted low Se status is eliciting protective and immune supportive health benefits, both in prevention and adjuvant therapy of the different AID and COVID-19, in particular for patients residing in areas of low habitual Se intake. From all we know today, there is no guarantee that supplemental Se will provide measurable health benefits to all patients, but the probability that a subset will profit from Se when provided as substitution is high. The avoidance of severe Se deficiency will not only reduce AID risk, but also confer some protection from other relevant diseases, including cancer, and cardiovascular and infectious diseases. There are no indications that Se substitution is associated with side effects if not applied as a supplement or as a drug of excessively high dosage, in particular when well-supplied subjects are excluded. Accordingly, the potential health benefits of supplemental Se are not of equal relevance and importance around the world, but largely restricted to areas with sub-optimal baseline supply, as reflected in the map shown above (Figure 2). However, even in areas of ample supply, severe diseases, such as sepsis or COVID-19, may cause immune-relevant Se deficiency irrespective of geography, and monitoring under such conditions is recommended (Table 2). First, this recommendation applies to critically ill patients in intensive care, irrespective of underlying disease. According to the aforementioned definition of substitution versus supplementation, there is at present no scientific rationale for Se supplementation, but substitution is mandatory in case of suspected or diagnosed Se deficiency, both for avoiding health risks (e.g., AID) and for supporting the immune system of an already or not-yet diseased organism.

## 7. Conclusions

The trace element Se is essential for a normal functioning of the immune system, and severe Se deficits impair immune responses and predispose to AID, as characterized best for AID of the thyroid gland. An active immune system with elevated cytokine levels exerts a suppressive effect on hepatic selenoprotein biosynthesis and SELENOP secretion into the circulation, causing reduced Se metabolism and an overall suppressed Se status. An acute or chronic severe Se deficit perturbs the immune system, irrespective of the underlying reason (e.g., infection, pregnancy, trauma, cancer, poor nutrition, surgery). Notably, the conditions causing low Se status overlap with known triggers for AID, and indicate a potential direct causal interrelation (Table 2). This interrelationship and the benefit of supplemental Se are firmly established for the thyroid, but not yet for the other prevalent AID, such as RA or T1DM. Consequently, avoiding severe Se deficits may reduce AID risks and alleviate disease symptoms, both in prevention and during therapy. Should this theory be verified by suitable RCT, the insight would be of broad medical relevance and contribute to a better understanding, control, and reduction of the elevated postpartum, post-infection, and post-injury AID risk. Accordingly, Se substitution is strongly recommended for chronic or acute deficiency, whereas supplementation to healthy subjects with sufficiently high baseline levels does not appear to be warranted.

## Figures and Tables

**Table 1:** Selenoproteins with particular functions in the immune system.

Glutathione peroxidases 1, 2 (GPX1, 2)	control intracellular peroxide tone	[160]
Glutathione peroxidase 4 (GPX4)	reduces lipid hydroperoxides, prevents ferroptosis	[161]
Iodothyronine deiodinases (DIO2, 3)	regulate activity of innate immune cells	[162]
Methionine-R-sulfoxide reductase (MSRB1)	reduces oxidized Met, affects F-actin formation	[163]
Selenoprotein H (SELENOH)	controls redox-sensitive transcription and damage	[164]
Selenoprotein I (SELENOI)	contributes to the biosynthesis of phospholipids	[165]
Selenoprotein K (SELENOK)	affects Ca-signaling and activity of lymphocytes	[166]
Selenoprotein P (SELENOP)	mediates hierarchical Se supply, indicates Se status	[8]
Selenoprotein S (SELENOS)	controls quality of proteins synthesized in ER	[149]
Selenoprotein T (SELENOT)	controls redox state and protein quality in ER	[144]
Selenophosphate Synthetase 2 (SEPHS2)	controls selenoprotein biosynthesis rate	[167]
Selenoprotein 15 (SEP15, SELENOF)	gatekeeper for ER exit of immunoglobulins	[168]
Thioredoxin reductases 1, 2 (TXNRD1, 2)	regenerate thioredoxin, balance mitochondrial ROS	[169]

**Table 2:** Inflammatory conditions associated with decreasing Se status and increasing AID risk.

Condition	Effect on Se Status	Reference *	Effect on AID Risk	Reference *
bacterial infection	suppression	[142]	enhancement	[196]
viral infection	suppression	[187]	enhancement	[197]
cancer	suppression	[198]	enhancement	[199]
(poly-)trauma	suppression	[200]	enhancement	[201]
burn injury	suppression	[202]	enhancement	[203]
smoking	suppression	[204]	enhancement	[205]
surgery	suppression	[206]	enhancement	[207]
transplantation	suppression	[190]	enhancement	[208]
pregnancy	suppression	[82]	enhancement	[85]

* out of many suitable references, one is chosen for reasons of space and clarity, with an apology to the authors of relevant studies not listed here. Admittedly, this overview is a biased selection intended to motivate and stimulate reflection.
